# *MAEWEST* Expression in Flower Development of Two *Petunia* Species

**DOI:** 10.3390/ijms140713796

**Published:** 2013-07-03

**Authors:** Ana Lúcia A. Segatto, Andreia Carina Turchetto-Zolet, Lilian Cristina B. Aizza, Carolina C. Monte-Bello, Marcelo C. Dornelas, Rogerio Margis, Loreta B. Freitas

**Affiliations:** 1 Laboratory of Molecular Evolution, Department of Genetics, Universidade Federal do Rio Grande do Sul, P.O. Box 15053, 91501-970 Porto Alegre, RS, Brazil; E-Mail: analuciasegatto@gmail.com; 2 Laboratory of Genomes and Plant Population, Center of Biotechnology and Department of Biophysics, Universidade Federal do Rio Grande do Sul, P.O. Box 15053, 91501-970 Porto Alegre, RS, Brazil; E-Mails: aturchetto@gmail.com (A.C.T.-Z.); rogerio.margis@ufrgs.br (R.M.); 3 Department of Plant Biology, Institute of Biology, Universidade Estadual de Campinas, P.O. Box 6109, 13418-970 Campinas, SP, Brazil; E-Mails: lcbaizza@gmail.com (L.C.B.A.); carolmontebello@yahoo.com.br (C.C.M.B.); dornelas@unicamp.br (M.C.D.)

**Keywords:** *MAEWEST*, *Petunia inflata*, *Petunia axillaris*, organ fusion, flower morphology

## Abstract

Changes in flower morphology may influence the frequency and specificity of animal visitors. In *Petunia* (Solanaceae), adaptation to different pollinators is one of the factors leading to species diversification within the genus. This study provides evidence that differential expression patterns of *MAWEWEST* (*MAW*) homologs in different *Petunia* species may be associated with adaptive changes in floral morphology. The *Petunia* × *hybrida MAW* gene belongs to the *WOX* (*WUSCHEL-*related homeobox) transcription factor family and has been identified as a controller of petal fusion during corolla formation. We analyzed the expression patterns of *P. inflata* and *P. axillaris MAW* orthologs (*PiMAW* and *PaMAW*, respectively) by reverse transcriptase polymerase chain reaction (RT-PCR), reverse transcription–quantitative PCR (qRT-PCR) and *in situ* hybridization in different tissues and different developmental stages of flowers in both species. The spatial expression patterns of *PiMAW* and *PaMAW* were similar in *P. inflata* and *P. axillaris*. Nevertheless, *PaMAW* expression level in *P. axillaris* was higher during the late bud development stage as compared to *PiMAW* in *P. inflata.* This work represents an expansion of petunia developmental research to wild accessions.

## 1. Introduction

The great morphological variation seen in flowers is associated with the different pollinators of each species. Changes in flower color and shape may cause changes in frequency and specificity of animal visitors, contributing to speciation [1]. Even a mutation in a single gene may have a central role in pollinator shifts [2,3].

**Figure 1:**
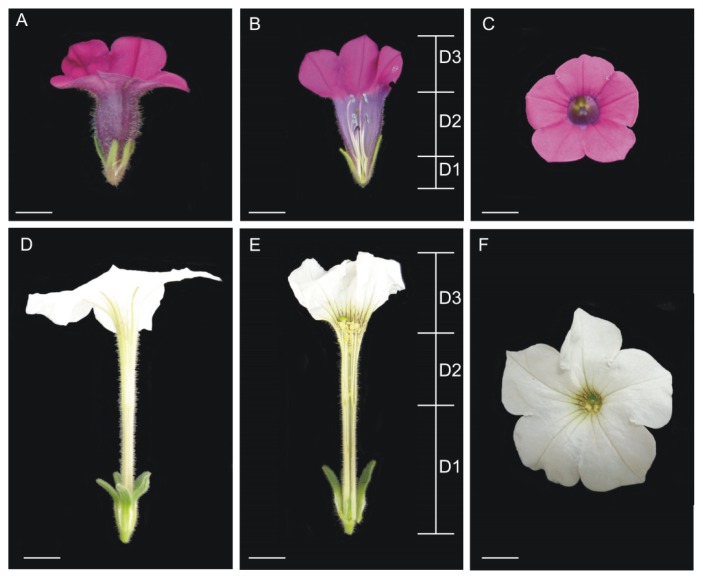
Morphological differences. (**A**–**C**) *Petunia inflata*; (**D**–**F**) *Petunia axillaris*; **A** and **D** flowers in side view; **B** and **E** flower internal view; with domains D1, D2 and D3 delimited [5]; **C** and **F** front view. Bars correspond to 1 cm.

Although it is known that some floral characteristics (such as those resulting from the fusion of floral parts, shape and size) arise after organ initiation, the developmental mechanisms associated with these processes are poorly understood [4]. Post-initiation fusion occurs in petals, carpels, and stamen filaments of *Petunia* flowers: the petal primordia partially fuse to form the corolla tube, the stamen filaments also fuse to the proximal part of the corolla tube, and the two carpel primordia fuse to form the pistil [5,6]. In addition, the flowers of the different species have distinct shape and size [6]. *MAEWEST* (*MAW*) gene is associated with organ fusion and lateral growth in *Petunia* × *hybrida* [7]*. MAW* belongs to the *WOX* (*Wuschel*-related homeobox) family of transcription factors and has functions that are partially similar to the *Arabidopsis thaliana* genes *WOX1* and *PRESEED FLOWER* (*PRS/WOX3*) involved in lateral organ development [8–11]. Accordingly, *maw* mutants showed defects in petal fusion, carpel fusion and lamina growth. Morphological analysis suggested that the phenotype could be the result of polarity defects along the adaxial/abaxial axis, which also affects leaves, bracts and sepals [7]. Here, we chose two wild *Petunia* species, *Petunia inflata* and *Petunia axillaris*, to analyze the expression patterns of their respective *MAW* orthologs. These are closely related species but nevertheless show great differences in their morphologies (Figure 1 and Table 1). *Petunia inflata* has a short and campanulate corolla tube, considered a small flower, while *P. axillaris* has a long and salveriform corolla tube and is a big flower. The differences in the size of the flower are pronounced and are considered to be adaptations to different pollinators. While *P. axillaris* is pollinated by nocturnal hawkmoths, *P. inflata* is pollinated by bees [12–14]. Until now, no gene involved in controlling the difference in flower shape and size between these two species has been identified. Expression patterns of *MAW* orthologs in both *Petunia* species were analyzed by using reverse transcriptase polymerase chain reaction (RT-PCR), reverse transcription-quantitative PCR (qRT-PCR), and *in situ* hybridization; these experiments were aimed at investigating whether *MAW* might be involved in determining flower morphological differences in these two species. Our results suggested an association between *MAW* orthologs expression and flower development in these *Petunia* species.

## 2. Results

### 2.1. Sequence Analysis

**Figure 2:**
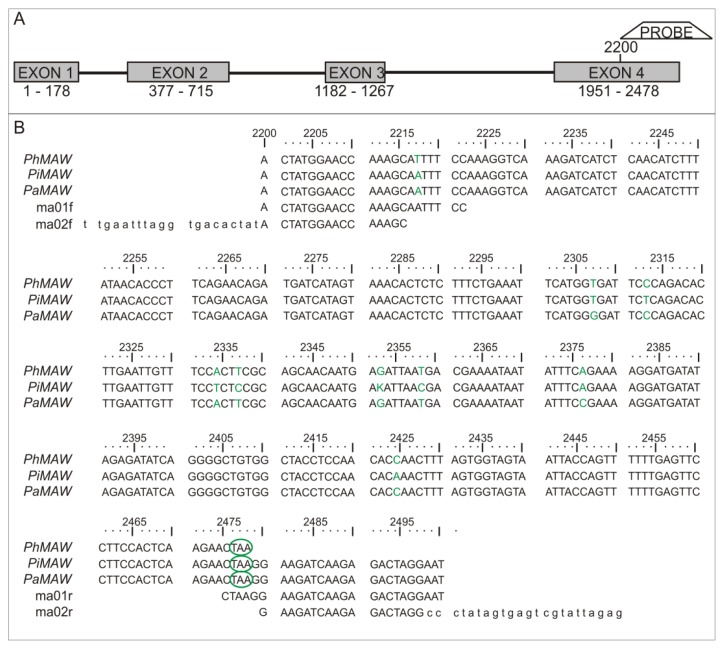
*MAWEWEST* (MAW) gene structure and alignment. (**A**) Gene structure and probe position in *Petunia × hybrida*; (**B**) *MAW* gene alignment. The numbers in **A**–**B** represent the base pair position in relation to the +1 frame. *PhMAW* is *Petunia × hybrida MAW* gene; *PiMAW* is *Petunia inflata MAW* gene; *PaMAW* is *Petunia axillaris MAW*; ma01f and ma01r refer to the first primer pair used; ma02f and ma02r refer to the second primer pair. Single nucleotide polymorphisms are represented in green, and green circles signify stop codons.

*Petunia* × *hybrida MAW* (*PhMAW*) orthologs were identified in wild *P. axillaris* and *P. inflata* and were denoted *PaMAW* and *PiMAW*, respectively. The *PaMAW* and *PiMAW* genomic sequences were deposited in GenBank as JQ438842 and JQ438843, respectively. The sequence similarities among *PaMAW*, *PiMAW* and *PhMAW* were obtained using BLASTX [15]. The sequence alignment between the *MAW* homologs described in this work is shown in Figure 2. Sequence similarity among the *P.* × *hybrida*, *P. inflata* and *P. axillaris MAW* homologs could be considered high (BLASTX *E*-values ≈ 4 × 10^−32^). Moreover, the phylogenetic analysis of *WOX* partial sequences showed orthology among *P. axillaris*, *P. inflata* and *P.* × *hybrida MAW* genes (Figures S1 and S2). Nine positions in a 301 bp region presented nucleotide mutations likely due to the recent diversification of the genus *Petunia*, though selective pressure cannot be ruled out.

### 2.2. RT-PCR and qRT-PCR

**Figure 3:**
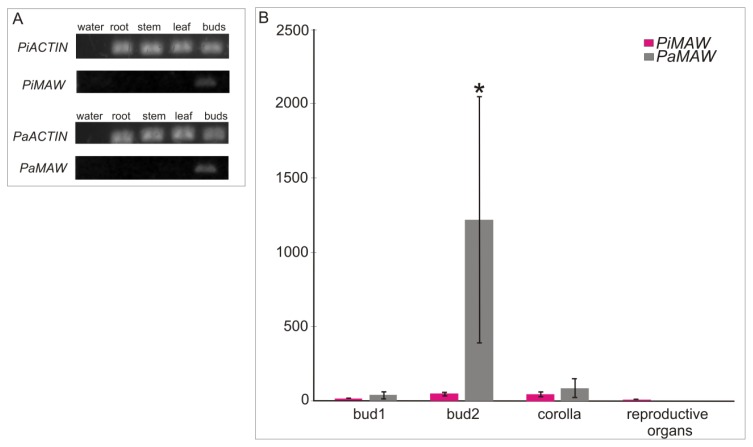
Gene expression analysis using RT-PCR and qRT-PCR. (**A**) Agarose gel electrophoresis of PCR amplification products of *MAW* transcripts from different floral organs of *Petunia inflata* and *Petunia axillaris*: mature root, stem and leaves, and floral buds; (**B**) Relative expression analysis of *MAW* transcripts present in *P. inflata* and *P. axillaris* by q-PCR. *P. axillaris* reproductive organs were used in calibrating the relative expression analysis among tissue types in both species. Relative expression was plotted using the *ACTIN* gene as a normalizer. Error bars represent the SEM of four biological and three technical repeats. *PiMAW* is *P. inflata MAW* gene expression; *PaMAW* is *P. axillaris MAW* gene expression; *PaACTIN* is *P. axillaris* and *PiACTIN* is *P. inflata ACTIN2* gene expression. Bud1: buds < 5 mm in length and Bud2: buds ≥ 5 mm in length. * significantly different means (*p* < 0.05).

*PiMAW* and *PaMAW* expression patterns are shown in Figure 3. According to the RT-PCR results, *MAW* transcripts were not detected in mature roots, stems or leaves (Figure 3A) but were detected in floral buds in both *P. axillaris* and *P. inflata* (Figure 3B). The use of qRT-PCR, a more sensitive and quantitative method, allowed for detection of a higher expression level of *PaMAW* in 5 mm or larger (bud 2) buds (Figure 3B). It is important to note that the high variance observed in the relative expression values among biological replicates. This kind of deviation is expected in real-time experiments [16], especially those using samples from natural populations that present high intrinsic genetic diversity. The expression level difference seen between the reproductive organs of the different species was not statistically significant due to this high variance (Figure 3B). In bud 2, the difference in *PaMAW* expression compared to *PiMAW* expression was statistically significant.

### 2.3. *In Situ* Hybridization

**Figure 4:**
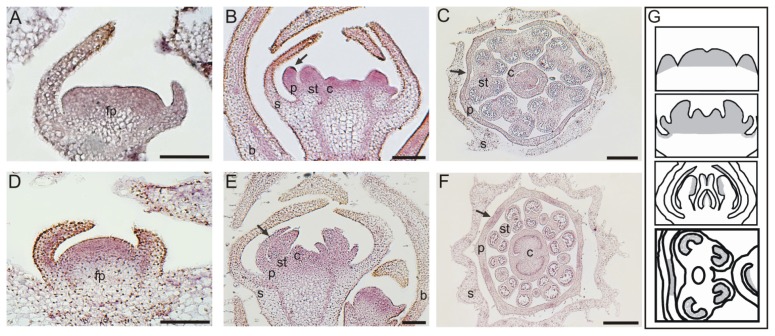
Gene expression analysis using *in situ* hybridization. The hybridization signal is detected as a purple precipitate. (**A**–**C**) *Petunia inflata* floral buds, **A** and **B**: longitudinal section of floral primordium, **C**: cross-section of late-developing stage; (**D**–**F**) *Petunia axillaris* floral buds, **D** and **E**: longitudinal section of floral primordium, **F**: cross section of late developmental stage of *P. axillaris* floral buds; (**G**) Schematic representation of gene expression pattern during flower development. The floral organ identities in **A**–**F** are as follows: fp, floral primordium; b, bract; s, sepal; p, petal; st, stamen; and ca, carpel. The arrow indicates the expression in petals. Bars correspond to 200 μm.

The local *PaMAW* expression pattern was similar to that observed for *PiMAW*, and both were similar to the expression pattern for *P.* × *hybrida MAW* [7]. During early floral meristem development, both *PaMAW* and *PiMAW* transcripts were detected in all emerging floral organ primordia (Figure 4A,D). As development proceeded, expression decreased in sepal primordia (Figure 4B,E) until it could no longer be detected (Figure 4C,F). In petal primordia, transcripts of both *PaMAW* and *PiMAW* were initially distributed uniformly (arrows in Figure 4B,E), but they became restricted to the distal ends of the primordia, corresponding to the points of organ fusion in later developmental stages (arrows in Figure 4C,F). The hybridization signal was also detected at the onset of stamen primordia and was detected only in stamen loculi at a later developmental stage (Figure 4C,F). Both *PaMAW* and *PiMAW* were evenly expressed in carpel primordia, and in later stages, the hybridization signal was stronger where the two carpels fused. Transcripts of both genes could also be detected in developing ovules (Figure 4C,F). The schematic representation in Figure 4G illustrates the stereotyped expression patterns of *PaMAW* and *PiMAW* during *Petunia* flower development, regions of concentrated expression are colored gray. This representation demonstrates that changes in *MAW* expression during the development progress becoming mainly restricted to regions where organ fusion occurs.

## 3. Discussion

The flower shape and size are considered defining characteristics for different pollinators in *Petunia*. Understanding the evolution of this trait may help us to elucidate the complex evolutionary history of the genus. Until now, no phylogenetic study could reliably clarify the ancestral and derived characteristics of *Petunia* species [6]. Speciation events must be related to the evolution of adaptations to different pollinators, and therefore, this is one potential path to unraveling the evolutionary history of the *Petunia* genus [3,17,18].

The broad expression of *MAW* orthologs detected by our RT-PCR, qRT-PCR and *in situ* hybridization in both *P. inflata* and *P. axillaris* flowers corroborates the role of this gene in the formation of reproductive organs, as in *P.* × *hybrida* [7]. While we detected expression of the *MAW* orthologs in *P. inflata* and *P. axillaris* in floral buds of sizes evaluated, *P. axillaris* showed a higher expression level later in flower development, indicating a possible connection between the differently sized flowers between these species. The *in situ* experiments confirmed the maintenance of *PaMAW* expression during later developmental stages of *P. inflata* and *P. axillaris* flowers, specifically in parts that underwent a process of post-initiation organ fusion as petals and carpels. In *P. × hybrida, PhMAW* expression was also detected in shoot/leaf primordia and in young leaves [7]. We did not detect expression of either *PiMAW* or *PaMAW* in any mature vegetative tissue we collected except the flower tissue. Lateral expansion of the five petal primordia and the formation of a ring structure results in petal fusion in *Petunia* [7]. During the first three stages of petal development in *Petunia*, the floral tube development occurs mainly by cellular division; from the fourth stage onward (floral buds with more than 4 mm), cell expansion predominates [19]. The corolla division in three structural domains is as follows: D1 is the part of the floral tube fused to the stamen filaments, D2 is the distal domain of the floral tube, and D3 is the limb [5] (Figure 1B,E). D1 is the main domain responsible for the differences in corolla tube length between *P. axillaris* and *P. inflata*. This region contains a larger number of cells in early developmental stages of *P. axillaris* and a larger cell length in mature flowers [5]. Thus, the difference in cell number occurs early in development while the difference in cell elongation occurs in later stages, and both processes contribute to corolla tube length. In the limb, the cell division persists to phases close the anthesis, and the cell elongation, which could be associated with flower opening, starts later [19]. In 5 mm or larger buds, the style and stigma are completely formed, the style is elongated and the ovule primordial arises [4]. Developmental defects were not detected in stamen in *maw* flowers [7].

The main difference found between *PaMAW* and *PiMAW* expression patterns was that *PaMAW* expression is higher at a later flower development stage. Organ fusion is characteristically affected in *P.* × *hybrida maw* mutants. While organ fusion occurs in both *P. axillaris* and *P. inflata*, the corolla of *P. axillaris* is bigger than that of *P. inflata. PaMAW* gene is more highly expressed in *P. axillaris* 5 mm or larger buds, a period of expression that coincided with cellular expansion in the corolla tube and cell division in the limb. It has been shown that growth in length and width are controlled by different mechanisms and *WOX* genes are involved in the organ lateral growth along the mediolateral axis, promoting cell proliferation [7–11,20–22]. The *MAW* orthologs may play a role in proper cell division and fusion and may participate in the developmental pathways that govern the differences in flower size between these two species. These initial findings indicate that more studies are necessary to establish the participation of these genes in the divergence of floral morphology between these species by investigating modified *MAW* expression patterns in transgenic *P. axillaris* and *P. inflata* plants. The spatial separation in the predominance of cell division or cell elongation in different moments of development in different domains of the corolla [5,19] must involve a mechanism of control and *MAW* gene expression should be modulated by this control mechanism. *WOX1* homologs of *Medicago truncatula*, *Nicotiana sylvestris* and *Pisum sativum* (*STENOFOLIA*, *LAM1*, and *LATHYROIDES* respectively) were described as required for cell division, acting in petal lobe expansion, controlling lamina elaboration in the mediolateral axis [20,21], which corroborates our proposition about the *MAW* homolgs in *Petunia*. Obviously, one cannot dismiss the hypothesis that additional genes might also participate in this process.

## 4. Experimental Section

### 4.1. Plant Material

*P. inflata* and *P. axillaris* seeds were collected from natural populations in Rio Grande do Sul, Brazil (27°46′S, 53°26′W and 30°36″S, 55°56′W, respectively) and were cultivated under greenhouse conditions (Biology Institute, UNICAMP, Campinas, SP, Brazil). Some plants were cultivated under hydroponic conditions to obtain root material for RNA extraction. Leaves were collected for genomic DNA extraction [23]. Floral buds in different developmental stages were collected, fixed in 4% paraformaldehyde, dehydrated in ethanol and xylene series and embedded in paraffin. Paraffin sections (8-μm-thick) were mounted in aminosilane-coated slides. Tissues from roots, shoots, leaves, and buds were collected in liquid nitrogen for RNA extraction. Floral buds and pre-anthesis flowers were also collected directly from natural populations and stocked in Trizol^®^ (Invitrogen, Carlsbad, CA, USA) until RNA extraction.

### 4.2. Sequencing and Riboprobe Labeling

The *Petunia* × *hybrida MAW* (*PhMAW*) coding region sequence (CDS) was obtained from GenBank (EU359004.1) and used as a query to find homologous sequences from *P. inflata* and *P. axillaris* in the *Petunia* EST database [24]. The obtained sequences were used to design specific primers using the Primer3 tool, version 0.4 [25]. The primers (ma01f and ma01r) were designed to anneal to a region corresponding to the 3′ end of the *PhMAW* coding sequence downstream from the region coding the conserved homeodomain, preventing cross-hybridization of the probes (Figure 2A,B). Due to the high similarity among the selected *Petunia* sequences in this segment, the same pair of primers was used to amplify fragments from both *P. inflata* and *P. axillaris*. PCR amplifications were performed in 25 μL reactions consisting of 1 U Taq polymerase (Invitrogen, Carlsbad, CA, USA), 1× buffer (Invitrogen, Carlsbad, CA, USA), 0.2 mM each dNTP, 0.2 mM MgCl_2_, 0.2 μM of each primer and 20–50 ng of genomic DNA as a template. Amplification conditions were as follows: 3 min at 94 °C, 30 cycles of 30 s at 94 °C, 30 s at 50 °C, and 40 s at 72 °C, with a final 5 min extension step at 72 °C. PCR products were purified using 20% polyethylene glycol [26] and sequenced in a MegaBACE 1000 DNA Analysis System (GE Healthcare, Biosciences, Pittsburgh, PA, USA) using the ET Terminator Kit (GE Healthcare, Biosciences, Pittsburgh, PA, USA) according to the manufacturer’s instructions. In order to verify if the sequences obtained are orthologs of *P.* × *hybrida MAW*, we conducted a phylogenetic analysis using *WOX*’s partial sequences proteins of *P.* × *hybrida*, *P. inflata*, *P. axillaris* and *Arabidopsis thaliana. WOX* sequences of *P. × hybrida* and *A. thaliana* were obtained from NCBI [27] and Phytozome [28], respectively. The *WOX* protein sequences were aligned using CLUSTALW as implemented in MEGA 5.1 [29] and manually adjusted. The phylogenetic analysis was performed using Neighbor-Joining in MEGA. The molecular distances of the aligned sequences were calculated according to the *p-*distance parameter with all gap and missing data accounted as pairwise deletion. Branch points were tested for significance by bootstrapping with 1000 replications. *In situ* probes were prepared by using a second primer pair (ma02f and ma02r) in a secondary PCR reaction that used the first PCR product as a template, adding the T7 and SP6 sequences to the first PCR product. The secondary PCR reaction was conducted under the same conditions as the first PCR. The probe was synthesized using the secondary PCR product, which was purified with the PureLink^®^ PCR Purification kit (Invitrogen, Carlsbad, CA, USA). Approximately 200 ng of the purified PCR product was used to synthesize the sense and antisense probes by *in vitro* transcription using the DIG RNA Labeling kit (Roche, Penzberg, Germany), which includes digoxigenin-labeled uracils (DIG-UTPs).

### 4.3. RNA Extraction, RT-PCR and qRT-PCR

Temporal RNA expression patterns of *MAW* orthologs from *P. inflata* and *P. axillaris* were analyzed in extracts from mature roots, stems, leaves, and floral buds (bulks of buds in different developmental stages: buds smaller than 5 mm and buds equal or larger than 5 mm). Total RNA was extracted using Trizol^®^ (Invitrogen, Carlsbad, CA, USA), and cDNA was synthesized using the Super Script First Strand Synthase kit (Invitrogen, Carlsbad, CA, USA). The *Petunia ACTIN* gene was used as a positive control for cDNA synthesis [7]. The cDNA samples were used as templates in PCR reactions with the ma01f and ma01r primer pair. Amplification conditions were as follows: 3 min at 94 °C followed by 30 cycles of 10 s at 94 °C, 10 s at 57 °C, and 20 s at 72 °C, with a final 5-min extension step at 72 °C. To confirm expression patterns, perform a quantitative analysis, and investigate the expression pattern in non-cultivated plants, we performed qRT-PCR on RNA extracted from buds of two different stages (smaller than 5 mm or from 5 to 25 mm) and pre-anthesis flowers collected in the field. Floral buds were separated into two groups according to length in order to associate gene expression to stages in which growth occurs predominantly by either cell division (floral buds smaller than 5 mm) or cell expansion (buds equal or larger than 5 mm) [19]. In the qRT-PCR experiment, the ma01f and ma01r primers were used. Relative transcript abundance was detected with the intercalating dye SYBR Green in a 7500 Real Time PCR System (Applied Biosystems, Foster City, CA, USA). Reactions were performed in a 20 μL final volume composed of 10 μL of cDNA sample previously diluted 1:100 in water, the specific primer-pairs and 10 μL of Sybr-Green PCR-mix (Ludwig Biotech, Alvorada, RS, Brazil). Amplification conditions were as follows: one initial cycle of denaturation at 95 °C for 5 min, followed by 40 cycles of 95 °C for 15 s 60 °C for 10 s and 72 °C for 15 s and data acquisition at 60 °C for 35 s. Melting curves from 55 to 99 °C were obtained to confirm the amplification of a single specific product of PCR. All reactions were performed using four independently isolated biological RNA samples and in three technical replications. *ACTIN* was used as a reference gene for sample normalization, and *P. axilaris* reproductive organs were used for calibrating calculations performed with the 2^−ΔΔCt^ method [30]. One-way ANOVA with the Duncan test (*p* ≤ 0.05) was applied in SPSS v.20 (IBM Corp, New York, NY, USA) to compare differential expression values between the different development stages of flowers and the tissues of species.

### 4.4. *In Situ* Hybridization

Hybridization was performed in floral buds in different developmental stages with non-radioactive probes [31]. The hybridization signal was detected by a colorimetric assay in which an anti-digoxigenin antibody coupled with alkaline phosphatase and NBT/BCIP (nitro blue tetrazolium chloride/5-Bromo-4-chloro-3-indolyl phosphate toluidine) served as a substrate. The results were documented using a ZEISS AXIOSKOPE microscope (Zeiss, Jena, Germany).

## 5. Conclusions

In this work, we used spatial and temporal expression patterns of *MAW* orthologs from two *Petunia* species to associate the well-described phenotype of the *P. × hybrida maw* mutant with the adaptation to different pollinators of contrasting *Petunia* wild species. We found evidence that this locus might be involved with the evolution of perianth characteristics in *Petunia.* The results suggest a possible role of this gene in the different flower size and shape between *P. axillaris* and *P. inflata*. In light of the great number of developmental mutants available that have been studied in model plants such as *P. × hybrida*, analyzing spatial and temporal expressions of genes in species with contrasting phenotypes associated to those genes would be a good approach to shed light on the putative molecular nature of developmental adaptations.

## Supplementary Information



## Figures and Tables

**Table 1:** Main floral differences between *Petunia axillaris* and *Petunia inflata*.

Characteristic	*Petunia axillaris*	*Petunia inflata*
Pollen	yellow	bluish
Petal color	white	purple
Filaments	adnated to the middle of the tube	adnated to the base of the tube
Corolla shape	hypocrateriform	funnelform
Self-compatibility	self-compatible/self-incompatible	self-incompatible
Nectar	large amounts	low amounts
Volatiles	large amounts	low amounts
Habit	erect	ascendant
