# Supplementary material for: MAEWEST Expression in Flower Development of Two Petunia Species
Source: Int J Mol Sci. 2013 Jul 3;14(7):13796–807. doi: 10.3390/ijms140713796 (PMC3742218; doi:10.3390/ijms140713796)
Supplement: Supplementary file 1 [file ijms-14-13796-s001.pdf]

Supplementary Information

Figure S1. Neighbor-Joining phylogenetic tree obtained in MEGA 5.1 using partial sequences of WOX protein, with 1000 bootstrap replications. Bootstrap values are indicated above the branches. Species names are abbreviated as: At, *Arabidopsis thaliana*; Ph, *Petunia* × *hybrid*; Pi, *Petunia inflata*; Pa, *Petunia axillaris*, and accession codes are provided following the protein names. *WOX1* group is indicated by a black box.

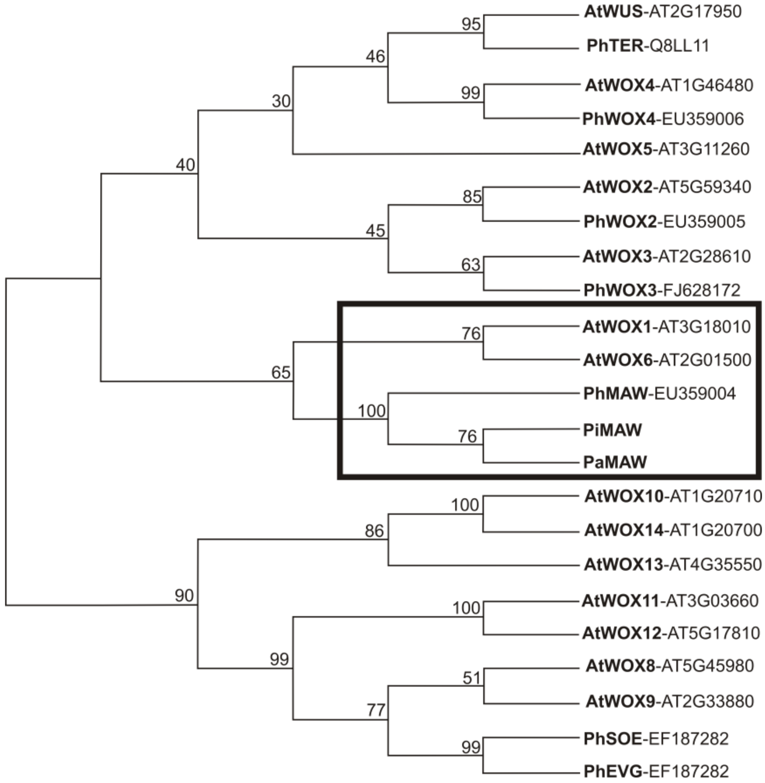

Figure S2. Alignment used to construct the Neighbor-Joining Tree. Conserved motifs *WUS* box [10] and *3'cWOX1/2* [8] are indicated by black boxes.

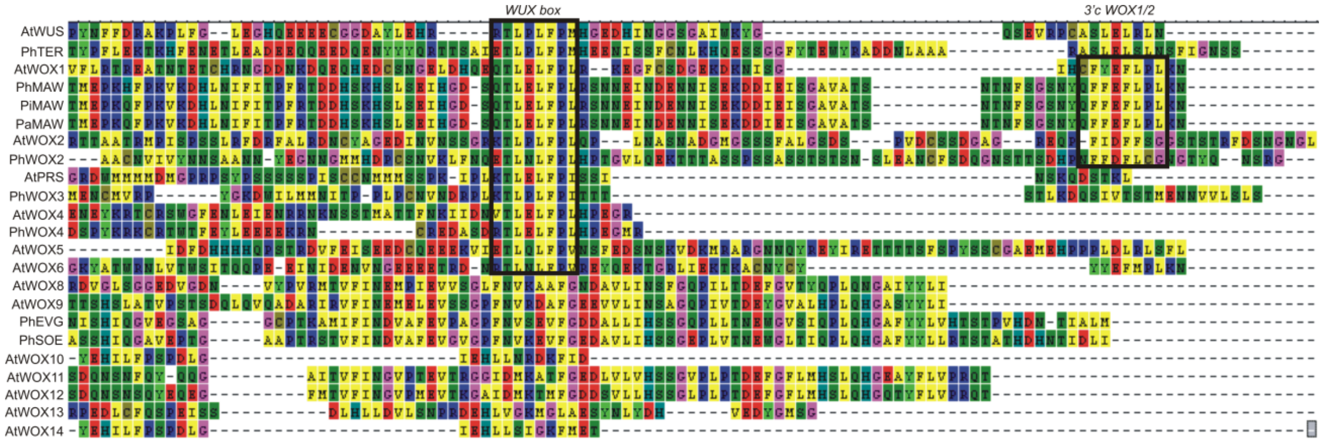

© 2013 by the authors; license MDPI, Basel, Switzerland. This article is an open access article distributed under the terms and conditions of the Creative Commons Attribution license (http://creativecommons.org/licenses/by/3.0/).
